# Standards and Guidelines for a New Decade

**DOI:** 10.1093/geroni/igab046.2132

**Published:** 2021-12-17

**Authors:** Tamar Shovali

**Affiliations:** Eckerd College, Eckerd College, Florida, United States

## Abstract

As the field evolved, so too, has the Gerontology and Geriatrics Curricular Standards and Guidelines in Higher Education. The 7th edition is focused on the integration of the highly vetted AGHE Gerontology Competencies for Undergraduate and Graduate Education. Through this work we establish a set of standards and guidelines that provides the foundation for excellence in gerontology and health professions education at various levels and across a range of programs in the US and globally. The document provide a model for institutions of higher education in the development of new programs, a basis for program assessment and evaluation, guidance for academic or institutional reviews in existing programs, and a platform for what constitutes a program of study in gerontology/geriatrics or health professions programs for students, the public, and employers. An overview of the development of the 7th edition and suggestions for its use will be provided.

